# Microbiomes in Soils Exposed to Naturally High Concentrations of CO_2_ (Bossoleto Mofette Tuscany, Italy)

**DOI:** 10.3389/fmicb.2019.02238

**Published:** 2019-10-04

**Authors:** Stefano Fazi, Fabrizio Ungaro, Stefania Venturi, Lara Vimercati, Carolina Cruz Viggi, Silvia Baronti, Francesca Ugolini, Costanza Calzolari, Franco Tassi, Orlando Vaselli, Antonio Raschi, Federico Aulenta

**Affiliations:** ^1^Water Research Institute, National Research Council (IRSA-CNR), Rome, Italy; ^2^Institute of BioEconomy – National Research Council (IBE-CNR), Florence, Italy; ^3^Institute of Geosciences and Earth Resources, National Research Council (IGG-CNR), Florence, Italy; ^4^Department of Earth Sciences, University of Florence, Florence, Italy; ^5^Department of Ecology and Evolutionary Biology, University of Colorado Boulder, Boulder, CO, United States

**Keywords:** mofette, CO_2_, bacteria, soil, acetogenesis

## Abstract

Direct and indirect effects of extremely high geogenic CO_2_ levels, commonly occurring in volcanic and hydrothermal environments, on biogeochemical processes in soil are poorly understood. This study investigated a sinkhole in Italy where long-term emissions of thermometamorphic-derived CO_2_ are associated with accumulation of carbon in the topsoil and removal of inorganic carbon in low pH environments at the bottom of the sinkhole. The comparison between interstitial soil gasses and those collected in an adjacent bubbling pool and the analysis of the carbon isotopic composition of CO_2_ and CH_4_ clearly indicated the occurrence of CH_4_ oxidation and negligible methanogenesis in soils at the bottom of the sinkhole. Extremely high CO_2_ concentrations resulted in higher microbial abundance (up to 4 × 10^9^ cell g^–1^ DW) and a lower microbial diversity by favoring bacteria already reported to be involved in acetogenesis in mofette soils (i.e., Firmicutes, Chloroflexi, and Acidobacteria). Laboratory incubations to test the acetogenic and methanogenic potential clearly showed that all the mofette soil supplied with hydrogen gas displayed a remarkable CO_2_ fixation potential, primarily due to the activity of acetogenic microorganisms. By contrast, negligible production of acetate occurred in control tests incubated with the same soils, under identical conditions, without the addition of hydrogen. In this study, we report how changes in diversity and functions of the soil microbial community – induced by high CO_2_ concentration – create peculiar biogeochemical profile. CO_2_ emission affects carbon cycling through: (i) inhibition of the decomposition of the organic carbon and (ii) promotion of CO_2_-fixation via the acetyl-CoA pathway. Sites naturally exposed to extremely high CO_2_ levels could potentially represent an untapped source of microorganisms with unique capabilities to catalytically convert CO_2_ into valuable organic chemicals and fuels.

## Introduction

Natural diffuse gas emitting areas, emanating almost pure volcanic or thermometamorphic CO_2_ to the atmosphere, commonly result in CO_2_ concentrations (>90% v/v) in the soil markedly higher than typical soil CO_2_ contents (ranging from near atmospheric levels to 100-fold higher values and generally <10% v/v; e.g., Amundson and Davidson, 1990; Oh et al., 2005; Schaetzl and Anderson, 2005), which are likely on par with CO_2_ concentrations in the Earth’s atmosphere when photosynthesis evolved (Beulig et al., 2016).

Soil biogeochemical processes in areas affected by direct and indirect influence of extremely high CO_2_ levels are poorly studied although they could potentially have significant ecological, environmental and biotechnological implications. Despite the fact that responses to high CO_2_ emissions were mainly studied on plants and on their capacity of carbon fixation, recent investigations clearly pointed out the pivotal role played by naturally occurring microbial communities on the utilization of volcanic CO_2_ and its incorporation into the soil organic matter (Beulig et al., 2016). Microbially driven CO_2_ utilization is a ubiquitous process in soils, carried out by different microbial metabolic pathways. Under chemical-physical conditions typical of venting spots (e.g., acidic pH and absence of oxygen), strictly anaerobic autotrophic prokaryotes, such as acetogenic bacteria (using hydrogen to reduce carbon dioxide into acetic acid) and hydrogenotrophic methanogenic archaea (using hydrogen to reduce carbon dioxide to methane), were reported to be the dominant members of the soil microbiome (Beulig et al., 2015). Both acetogens and methanogens use molecular hydrogen as electron donor and CO_2_ as terminal electron acceptor in their energy metabolism, producing acetate or methane as end-products. Several environmental parameters (e.g., temperature, pH, hydrogen partial pressure) influence competitive interactions between these two trophic groups, ultimately shaping the flow of carbon and electrons, since either acetate or methane can be produced. At low temperatures (i.e., <15°C) and acidic pH (i.e., <5), acetogenic bacteria can outcompete methanogens for hydrogen use, due to their higher growth rates (Cord-Ruwisch et al., 1988; Kotsyurbenko et al., 2001). By contrast, due to their higher affinity for hydrogen, documented by a lower half-saturation Michaelis-Menten constant and lower minimal hydrogen threshold concentration, methanogens can have a competitive advantage over acetogens in environments characterized by a low hydrogen availability (Cord-Ruwisch et al., 1988; Kotsyurbenko et al., 2001). To date, the competition between methanogens and acetogens was primarily investigated in laboratory-scale bioreactors typically operating under highly controlled, steady-state conditions, whereas only limited information is available on its relevance in natural ecosystems.

Sites naturally exposed to extremely high CO_2_ levels could potentially represent an untapped source of microorganisms with unique capabilities to catalytically convert CO_2_ (at levels as high as those typically occurring in flue gasses emissions of industries or even anaerobic digestion plants) into valuable organic chemicals and fuels. Such microorganisms have recently received attention in the context of microbial electrosynthesis, i.e., a novel technology in which electric current, ideally produced from renewable energy sources (e.g., solar, wind), is supplied to living microorganisms via a cathode to reduce CO_2_ to yield industrially relevant products, such as methane, volatile fatty acids, and alcohols (Rabaey and Rozendal, 2010). One of the most attractive aspects of microbial electrosynthesis is the remarkable conversion efficiency (> 80%) of electricity to chemicals (e.g., acetate). On the other hand, the relatively low production rates and costly product separation processes reported so far, still largely challenge the commercial exploitation of the technology.

Microbial processes at extremely high CO_2_ soil concentration levels are also relevant within the broader context of carbon capture and storage (CCS) technologies. The urgent need for large-scale solutions to reduce atmospheric levels of greenhouse gasses has indeed prompted the interest toward CO_2_ storage in deep saline aquifers or exhausted gas and oil reservoirs. However, before underground CO_2_ storage can be implemented at large scale, it is necessary to determine the potential environmental risks associated with the leakage of CO_2_ from the reservoir to the near surface environment, to minimize possible environmental impacts. In principle, leakage of large volumes of CO_2_ can remarkably affect structures and functions of soil microbial communities, and the overall biogeochemical processes they mediate (McFarland et al., 2013). Sites characterized by CO_2_-rich gas discharges represent unique natural analogs of engineered carbon storage sites experiencing steady CO_2_ leakage events. These sites could therefore provide the unique opportunity for studying the potential impacts of CO_2_ on near-surface ecosystems and groundwater and developing monitoring strategies and possibly preventing CO_2_ leakage events.

Natural CO_2_-rich reservoirs commonly occur worldwide (Pearce et al., 2004; Pearce, 2006) and their distribution is mainly controlled by Cenozoic rift systems, e.g., East African Rift System (Vaselli et al., 2002; Smets et al., 2010), Tertiary volcanism and hydrothermal and volcanic systems related to both Quaternary to recent volcanic activity and sedimentary basins.

The Bossoleto hydrothermal area at Rapolano (Tuscany, Italy), where fluxes of gas originated by both thermometamorphic processes on limestone and mantle degassing flow up to the surface in correspondence of deep fractures connected to the faults system (Minissale et al., 2002; Guerra and Raschi, 2004; Brogi et al., 2007), represents a typical example of natural CO_2_ storage site.

The objective of this study was to investigate the specific interactions between soil CO_2_ concentration changes and the microbial community dynamics in terms of structure and function to improve our understanding of microbially mediated C cycle, including the potential impact of CO_2_ on near-surface ecosystems. Onsite investigations in natural CO_2_ vents were integrated with laboratory experimental approaches in order to: (i) examine the diversity of soil microbial communities (both bacteria and archaea) in natural environments with extremely high CO_2_ concentration by 16S rRNA gene sequencing and by *in situ* hybridization approach (CARD-FISH) and (ii) use laboratory incubations to test the acetogenic and methanogenic potential of the microbial communities differently exposed to natural CO_2_ enrichment.

## Materials and Methods

### Site Description and Soil Sampling Strategy

**FIGURE 1:**
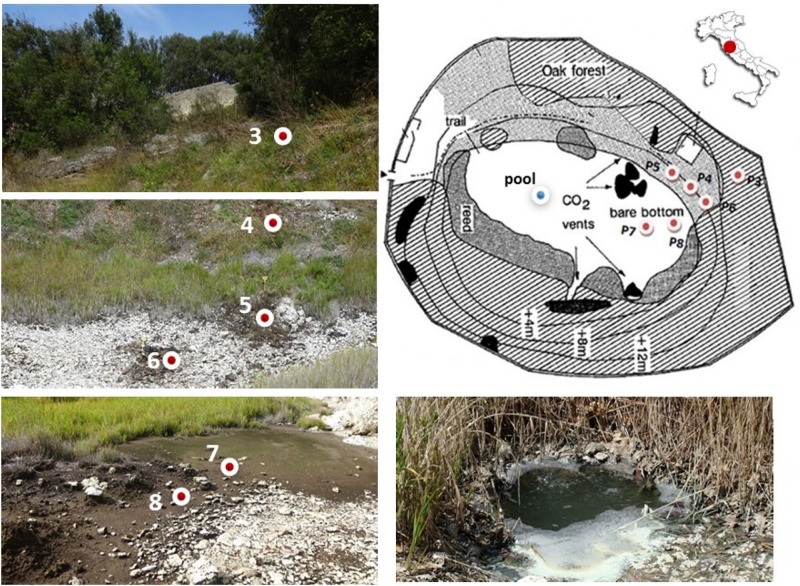
Sampling sites along the geomorphological gradient of the Bossoleto mofette (Rapolano, Tuscany, Italy) (Photos by S. Fazi, Site map modified after Körner and Miglietta, 1994).

The Bossoleto mofette belongs to the Rapolano hydrothermal area (Figure 1). It is a round shaped sinkhole characterized by gaseous emissions primarily consisting of CO_2_. In this sinkhole, a CO_2_ lake forms every night due to the combination of site topography and CO_2_ accumulation. Typically, CO_2_ concentrations range from 0.04 to 80% (v/v) over a 24-h period. During the night, the concentrations build up, reaching a maximum of about 80% at around 7:00 AM; afterward (typically after 9:00 AM), a rapid decrease occurs when direct radiation is incident on the bottom of the mofette (Van Gardingen et al., 1995; Kies et al., 2015). Due to infrared absorption by CO_2_, the temperature inside the sinkhole can be up to 30°C, i.e., higher than the corresponding air temperature (Kies et al., 2015). Inside the Bossoleto area, plant populations have therefore existed at elevated CO_2_ for hundreds of years (Miglietta et al., 1993; Körner and Miglietta, 1994; Raschi et al., 1997), an ample time for the occurrence of evolutionary changes in microorganisms with short generation times (Collins and Bell, 2006).

Soil sampling was carried out at six locations along a geomorphological gradient spanning from the peak of the sink-rim (site 3), to the back-slope (sites 4-5-6) and to the toe-slope at the bottom of the sinkhole (sites 7-8) (Figure 1). At each sampling site in the sink-rim and at the back-slope, soil samples were collected from the topsoil (0–10 cm, named “Sur”) and the subsurface layers (35–45 cm, hereafter named “Sub”). At the bottom of the sinkhole in sites 7 and 8 the “Sub” samples were collected at 20–30 cm depth. Only at site 7 an additional subsurface sample was collected at −60 cm depth (hereafter named “Deep”). A free-gas sample was collected from one of the bubbling pools (named “BP”) located at the bottom of the Bossoleto sinkhole close to the soil gas sampling sites.

### Soil Analyses

In three selected sites (site 3, site 4 and site 7 - Figure 1), soils were characterized in both the field and laboratory from the surface to a depth of approximately 60 cm depth, for a number of parameters including pH, HCl reaction, micro- and macro-nutrients and C-N content. Soil horizons were identified, described and sampled in the field following standard soil survey protocol (USDA, 2017). The pH was measured in a soil/water suspension (1:2.5 mass ratio). HCl reaction was observed in the field by using a 1M solution for determining effervescence class as a relative index of the carbonate amount in the soil matrix (USDA, 2017). Soil C and N contents were measured in laboratory using a CHN Elemental Analyzer (Carlo Erba Instruments, mod 1500 series 2). Dry sub-samples were also digested with a microwave oven (CEM, MARSXpress) according to the EPA method 3052 (U. S. Environmental Protection Agency, 2004). The solutions obtained after the mineralization were filtered (0.45 μm PTFE) and diluted. Micro- and macro-nutrients were determined by an ICP optical spectrometer (Varian Inc., Vista MPX) using scandium as internal standard.

### Gas Sampling and Analysis

Interstitial soil gasses were sampled from 5 sites (i.e., sites 3, 4, 5, 6 and 8) along vertical profiles from −10 to −50 cm, at regular depth intervals of 10 cm, except at site 8 where the maximum sampling depth was 20 cm, due to the presence of a shallow water table (Figure 1). Gas sampling was carried out using a stainless-steel tube (internal diameter: 0.4 cm) as described in Tassi et al. (2015). The gas from the pool was collected in a pre-evacuated glass vial (60 cm^3^) equipped with a thorion© valve and containing 20 mL of 4 M NaOH (Giggenbach, 1975). During sampling, CO_2_ and H_2_S dissolved in the alkaline solution, whereas non-condensable gasses (N_2_, O_2_, Ar, CH_4_, C_2_H_6_) were stored in the flask headspace. A glass vial for the analysis of ^13^C/^12^C ratios in CO_2_ (δ^13^C-CO_2_) was also collected.

Inorganic gasses in both vials (CO_2_, H_2_S, N_2_ and O_2_ + Ar) and the flask headspace (N_2_ and O_2_ + Ar) were analyzed using a Shimadzu 15A gas chromatograph (GC) and a Thermo Focus gas chromatograph, was used to separate the Ar and O_2_ peaks, as described in Tassi et al. (2015). CH_4_ and C_2_H_6_ were analyzed using a Shimadzu 14A gas chromatograph equipped with flame ionization detector (FID) and a 10 m stainless steel column packed with 80/100 mesh Chromosorb PAW coated with 23% SP 1700 as described in Tassi et al. (2015). The δ^13^C-CO_2_ values were measured by a Finnigan MAT 252 mass spectrometer after the extraction and purification of the gas mixtures (Evans et al., 1998; Vaselli et al., 2006). The δ^13^C-CH_4_ were analyzed using MS (Varian MAT 250) according to the procedure reported by Schoell (1980).

### Bacteria and Archaea Abundance and Diversity

The total bacteria and archaea cell abundances were assessed by Catalyzed Reported Deposition-Fluorescence *in situ* Hybridization (CARD-FISH) following extraction and detection procedures described elsewhere (Fazi et al., 2007; Amalfitano and Fazi, 2008). Subsamples of the purified cell suspension (1 mL) were filtered onto 0.2 μm-pore size polycarbonate membranes (47 mm diameter) and frozen at −20°C until analysis. Filter sections were then cut and hybridized in duplicate by rRNA-target HRP-labeled probes (Biomers, Ulm, Germany) targeting most Bacteria (EUB338, EUB338-II, EUB338- III) and Archaea (ARCH 915). The stained cells were quantified using an epifluorescence microscope (Leica, DM LB 30). At least 300 cells were counted in each filter section. Data are expressed as a percentage of total DAPI stained prokaryotes cells (%) and as abundance (cells/g of DW).

Sequencing approaches were employed to study microbial diversity across the environmental gradient. Samples of fresh soil from each site at two depths (three in site 7) were frozen directly in the field in dry ice and kept at −80°C until DNA extraction. DNA was extracted from each sample using the approach described in Fierer et al. (2012). To determine the diversity and composition of the bacterial communities, the protocol described in Leff et al. (2015) for amplicon 16S rRNA gene sequencing was used. Specifically, the 16S rRNA gene from isolated DNA was PCR-amplified using the 515f/806r primer pair. To prepare amplicons for sequencing, amplicon purification and normalization was done with Invitrogen SequalPrep Normalization Kit (Invitrogen Inc., CA, United States). Amplicons were combined into a single pool and sequenced using the Illumina MiSeq platform (BioFrontiers Institute, Boulder, CO, United States) using pair-end 2 × 150 bp chemistry.

### Data Processing and Statistical Analysis

Forward-oriented sequences were demultiplexed, quality filtered and processed using the Quantitative Insights into Microbial Ecology (QIIME) v1.9.1 bioinformatics package (Caporaso et al., 2010b). 16S rRNA gene paired-end reads were joined and singletons were excluded from further analysis and sequences with >97% identity were clustered into an OTU via UCLUST (Edgar, 2010). Representative sequences for each OTU were chosen for classification and the Greengenes 13.5 database (DeSantis et al., 2006) was employed to assign taxonomy identification to each single OTU. Based on this classification, all mitochondrial and chloroplast OTUs based on this classification were removed from the bacterial data set. The taxonomic assignments of the top OTUs from each treatment were verified by using BLAST to search NCBI, and refined as needed. Sequences were aligned with the PyNAST (Caporaso et al., 2010a) and a phylogeny was built with the FastTree algorithm (Price et al., 2009). OTU tables were rarefied to the lowest number of sequences in the lowest populated sample to make more robust comparisons and were used to assess alpha diversity and relative abundance of all taxa. A community-level Bray-Curtis distance matrix was generated and analyzed with a permutational multivariate analysis of variance (PERMANOVA) using an ADONIS model (Oksanen et al., 2013) to partition the variance in community composition. Principal Coordinate Analysis (PCoA) ordination was constructed based on the basis of the Bray-Curtis distance matrix and Hellinger transformed in order to visualize differences in community composition between low and high CO_2_ concentrations. Similarity percentage analysis (SIMPER) was used to determine the OTUs that contributed most to the observed dissimilarity between communities of low and high CO_2_ concentrations. All statistical tests, unless otherwise stated, were performed using the “vegan” R package (Oksanen et al., 2013).

The chemical variables were incorporated into a Non-metric MultiDimensional Scaling (NMDS) ordination plot in order to graphically synthesize the Bray-Curtis dissimilarity among samples. Chemical and microbial data were then projected onto the NMDS ordination using a vector-fitting procedure, in which the length of the arrow is proportional to the correlation between NMDS axes and each variable. This method allowed determining the variation pattern of each projected variable discriminating the samples (Foulquier et al., 2013; Amalfitano et al., 2014).

The 16S rRNA gene sequences from Illumina MiSeq libraries from this study were deposited in the SRA (Short Read Archive) database under Bioproject ID PRJNA548940.

### Assessment of the Acetogenic and Methanogenic Potential

To evaluate the acetogenic and methanogenic potential of the mofette microbial communities, a set of anoxic incubations was set up. Incubations were prepared in 120 mL (total volume) serum bottles, incubated statically, in the dark, at room temperature (20–25°C). Each bottle contained about 10 g (wet weight) of mofette soil (site 7sup, 7sub, 7deep, and site 3sub) and 40 mL of anaerobic mineral medium. The medium contained the following components: NH_4_Cl (0.5 g/L), MgCl_2_ × 6H_2_O (0.1 g/L), K_2_HPO_4_ (0.4 g/L), and CaCl_2_ × 2H_2_O (0.05 g/L). Upon preparation, all bottles were sealed with Teflon-faced butyl rubber stoppers, flushed with a N_2_/CO_2_ (70:30 v/v) gas mixture. H_2_ was added to half of the bottles to reach a final headspace concentration of 60:15 (v/v) H_2_:CO_2_. Upon setup, the pH value was in the range 5.5–6.0, hence close to typical pH values measured in the field.

Once all the bottles completely converted the initial dose of H_2_ (1st feeding cycle), they were flushed with the N_2_/CO_2_ gas mixture and then re-spiked with another dose of H_2_ (2nd feeding cycle). At the end of the first feeding cycle, the liquid volume cumulatively removed during incubation (i.e., for organic acids and pH analyses) was replaced with freshly prepared anaerobic medium. Each incubation experiment was set up in duplicate to ensure reproducibility.

Organic acids were analyzed by injecting 1 μL of filtered (0.22 μm porosity) liquid sample into a PerkinElmer Auto System gas-chromatograph equipped with a Flame Ionization Detector (FID). Gasses (H_2_, CH_4_ and CO_2_) were analyzed by injecting 50 μL of headspace sample into a Perkin-Elmer Auto System gas-chromatograph, equipped with a Thermal Conductivity Detector (TCD).

The percentage of reducing equivalents from the electron donor (i.e., H_2_) used in acetogenesis or methanogenesis was calculated at the end of each incubation from the measured levels of acetate and methane formed and the electron donor consumed. Molar equivalents factors used were: 8 eq/mol for acetate, 8 eq/mo1 for methane, and 2 eq/mol for hydrogen.

## Results

### Soil Characterization and Geochemical Analysis

**FIGURE 2:**
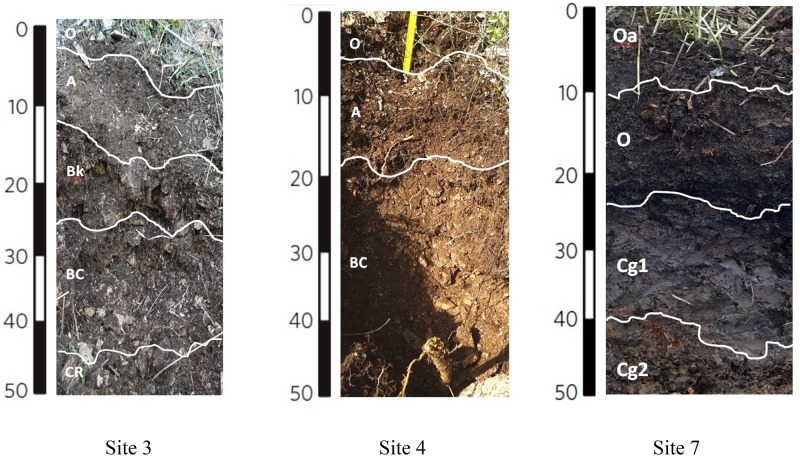
Soil profiles for sites 3, 4 and 7. The sequence of soil horizons along each soil profile is reported. Capital letters designate the master horizons and layers (O, horizons or layers dominated by organic soil materials; A, mineral soil horizon or layers at the soil surface or below an O horizon; B, subsurface mineral horizons that typically formed below an A or O horizon; C, subsurface mineral horizons or layers, excluding strongly cemented and harder bedrock, that are little affected by pedogenic processes and lack properties of O, A, and B horizons; R, strongly cemented to indurated bedrock). Two capital-letter symbols are used for transitional horizons, dominated by properties of one master horizon but have subordinate properties of another. Lowercase letters are used as suffixes to indicate specific characteristics of master horizons and layer (a, highly decomposed organic material; k, accumulation of carbonates; g, strong gleying due to saturation with stagnant water). Arabic numbers as suffixes indicate vertical subdivisions within a horizon or layer.

Soils from the Bossoleto sinkhole represent a toposequence with some typical aspects of soil formation in semiarid Mediterranean environments developed on travertine parent materials. Shallow skeletal soils are more developed on the summit of the sinkhole (site 3; 270 m a.s.l.) under oak forest (*Quercus ilex* L*.; Quercus pubescens* L*.; Fraxinus ornus* L.) and scanty herbaceous vegetation cover (*Cyclamen repandum* Sibth & Sm.; *Festuca inops* De Not., *Teucrium chamaedrys* L.). Under these conditions, a thick litter develops and the soil is characterized by the presence of a Bk horizon (i.e., with accumulation of calcium carbonate). The horizons sequence observed in the field is O-A-Bk-BC-Cr (Figure 2) and the soil is classified as Typic Calcixerept according to Soil Taxonomy (USDA, 2010) or as Skeletic Haplic Calcisol (IUSS Working Group Wrb, 2015). Soils along the slope are shallower and less developed: in the back-slope position (site 4; 264 m a.s.l) with uneven grass cover (*Sanguisorba minor* Scop., *Plantago lanceolata* L., *Centaurea deusta* Ten. subsp. deusta), water erosion is more active and only a less developed transitional BC horizon is observed along the soil profile. The horizons sequence observed in the field is O-A-BC-Cr and the soil is classified as (Lithic) Typic Xerorthent (USDA, 2010) or as a Skeletic Regosol (humic) (IUSS Working Group Wrb, 2015). The bottom of the sinkhole (site 7; 258 m a.s.l.) has scant herbaceous cover (*Agrostis stolonifera* L., *Phragmites australis* (Cav.) Trin. ex Steud.). Soil profile is formed on colluvial materials whose development is strongly affected by: (i) the rate of delivery of organic and mineral materials from the slopes of the sinkhole, (ii) the presence of high CO_2_ concentrations and (iii) the shallow fluctuating groundwater. Under these conditions, organic matter accumulates at the top of the soil profile (Oa and O organic topsoil horizons) and strongly anaerobic conditions lead to the formation of deeper Cg horizons with undecomposed root materials. The soil is classified as Thapto-Histic Fluvaquents (USDA, 2010) or Histic Gleyic Fluvisol (IUSS Working Group Wrb, 2015). Soil pH (field, lab and HCl reaction) exhibited a clear decrease along the toposequence (range 4.68–7.60) due to increasing CO_2_ concentrations in the pedosphere (along soil depth and along the toposequence), which resulted in the complete exploitation of the soil buffer capacity despite the calcareous nature of the parent material (Table 1). In terms of macro-nutrients such as C and N, the soil in site 3 showed intermediate content of N with a positive trend toward the bottom of the sinkhole (range 0.08–1.46%). Sites 3 and 7 also recorded high content of total C (range 2.31–16.61%), with site 5 showing the highest C:N ratio (range 10.32–149.02).

**TABLE 1:** pH, HCl reaction and C-N content in soil from Bossoleto mofette.

		**pH**	**HCl reaction**	**N%**	**C%**	**C/N**
Site 3	Sup	7.32	Strong	0.52	14.23	27.61
	Sub	7.31	Strong	0.25	10.98	44.03
Site 4	Sup	7.31	Strong	0.75	11.69	15.50
	Sub	7.60	Strong	0.29	10.14	35.46
Site 5	Sub	7.14	Strong	0.08	11.89	149.02
Site 6	Sub	6.68	Strong	0.49	12.88	26.33
Site 7	Sup	4.68	None	1.46	15.11	10.32
	Sub	5.76	None	0.16	2.31	14.54
	Deep	5.83	None	0.25	4.87	19.41
Site 8	Sup	5.36	None	1.38	16.61	12.05
	Sub	5.76	None	0.61	7.77	12.68

In terms of micro- and macro-nutrients (Supplementary Table 1), a loss of Ca (−93%) and other bases (e.g., Mg, Li) was observed in site 7 with respect to both sites 3 and 4. This was coupled with an increase in total P (+ 604%) and K (+ 113%) and Cu, Pb, Fe, Al and other microelements, likely because of the effect of the pH on element mobility.

### Chemical Composition of Gasses and δ^13^C-CO_2_ and δ^13^C-CH_4_ Values

The chemical composition (CO_2_, N_2_, H_2_S, O_2_, Ar in mmol/mol; CH_4_ and C_2_H_6_ in μmol/mol) and the δ^13^C-CO_2_ and δ^13^C-CH_4_ (in ‰ vs. V-PDB) of both soil gasses and bubbling pool is reported in Table 2. Interstitial soil gasses from profiles at sites 3, 4 and 5 were mainly consisting of N_2_ (from 744 to 821 mmol/mol) with variable concentrations of O_2_ (from 19 to 183 mmol/mol), CO_2_ (from 9.0 to 226 mmol/mol) and minor amounts of Ar (from 10 to 13 mmol/mol). Methane and C_2_H_6_ concentrations were up to 16 and 6.1 μmol/mol, respectively, whereas no H_2_S was detected (<0.05 mmol/mol). Sites 3, 4 and 5 were hereafter named LC: Low CO_2_ concentration sites.

**TABLE 2:** Chemical and isotopic composition of interstitial soil gasses and bubbling gasses (pool) from Bossoleto mofette.

**Site**	**Depth**	**CO_2_**	**H_2_S**	**N_2_**	**O_2_**	**Ar**	**CH_4_**	**C_2_H_6_**	**δ^13^C-CO_2_**	**δ^13^C-CH_4_**
										
	**cm**	**mmol/mol**	**mmol/mol**	**mmol/mol**	**mmol/mol**	**mmol/mol**	**μmol/mol**	**μmol/mol**	**‰ vs. V-PDB**	**‰ vs. V-PDB**
**Slope peak of the sink-rim**										
3	10	43	<0.05	799	147	11	0.5	0.5	–10.79	Nd
	20	67	<0.05	788	133	11	1.4	1.2	–10.11	nd
	30	101	<0.05	805	84	11	1.9	2.0	–11.50	nd
	40	143	<0.05	802	45	10	3.0	2.7	–8.50	nd
	50	183	<0.05	783	24	11	5.0	2.9	–7.82	nd
**Back-slope**										
4	10	9	<0.05	798	183	10	<0.1	<0.1	nd	nd
	20	11	<0.05	812	166	11	<0.1	<0.1	–10.45	nd
	30	13	<0.05	820	155	12	<0.1	<0.1	nd	nd
	40	17	<0.05	821	149	13	0.5	0.5	–11.87	nd
	50	18	<0.05	821	148	13	1.1	1.0	nd	nd
5	10	64	<0.05	820	106	10	1.6	2.0	–8.64	nd
	20	96	<0.05	804	89	12	2.4	2.0	–7.64	nd
	30	137	<0.05	814	38	11	5.0	4.4	–7.65	nd
	40	173	<0.05	791	25	12	11	5.9	–7.45	nd
	50	226	<0.05	744	19	12	16	6.1	–7.13	nd
6	10	397	<0.05	568	27	8.4	21	9.0	–7.10	nd
	20	404	<0.05	567	20	8.9	26	9.5	–7.83	−23.3
	30	373	<0.05	601	12	14	28	11	–8.05	nd
	40	670	<0.05	322	<0.5	8.3	18	6.3	–7.20	nd
	50	730	0.10	264	<0.5	6.5	19	5.9	–8.14	nd
**Toe-slope at the bottom of the sinkhole**										
7-8	10	805	0.11	190	<0.5	4.9	41	4.5	–6.06	nd
	20	807	0.18	188	<0.5	4.6	41	4.2	–6.38	−31.7
**Pool**		983	0.25	16	<0.5	1.1	125	7.9	–6.41	−38.5

The soil gasses from profile at site 6 had CO_2_ and N_2_ at comparable concentrations (up to 601 and 730 mmol/mol, respectively), relatively low O_2_ (≤27 mmol/mol), with Ar from 6.5 to 14 mmol/mol, and CH_4_ and C_2_H_6_ concentrations (up to 28 and 11 μmol/mol, respectively) slightly higher than those measured at sites 3, 4 and 5. At the maximum depth of profile at site 6, CO_2_ reached a concentration of 730 mmol/mol and H_2_S was detected (0.10 mmol/mol). Soil gasses from profile at sites 7-8 were characterized by dominant CO_2_ (up to 807 mmol/mol), whereas N_2_ and Ar concentrations were up to 190 and 4.9 mmol/mol, respectively. Oxygen was below the detection limit (<0.5 mmol/mol), H_2_S (up to 0.18 mmol/mol) and CH_4_ and C_2_H_6_ were up to 41 and 4.5 μmol/mol. Sites 6sub, 7 and 8 were hereafter named HC: High CO_2_ concentration sites.

**FIGURE 3:**
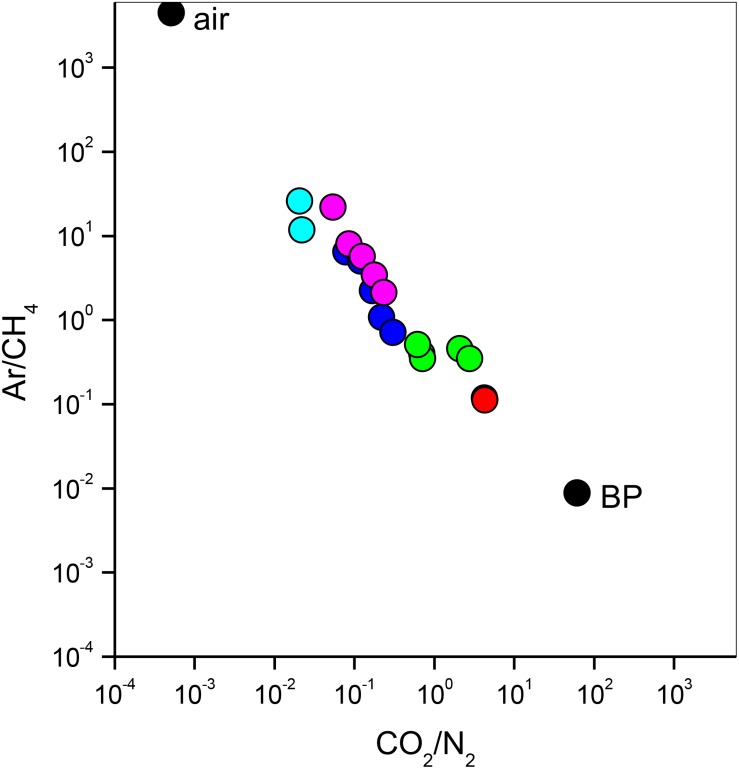
Ar/CH_4_ vs. CO_2_/N_2_ binary diagram for the interstitial soil gasses from site 3 (magenta circles), site 4 (cyan circles), site 5 (blue circles), site 6 (green circles) and site 8 (red circles). Air and gas from the bubbling pool (BP) are also reported (black circles).

**FIGURE 4:**
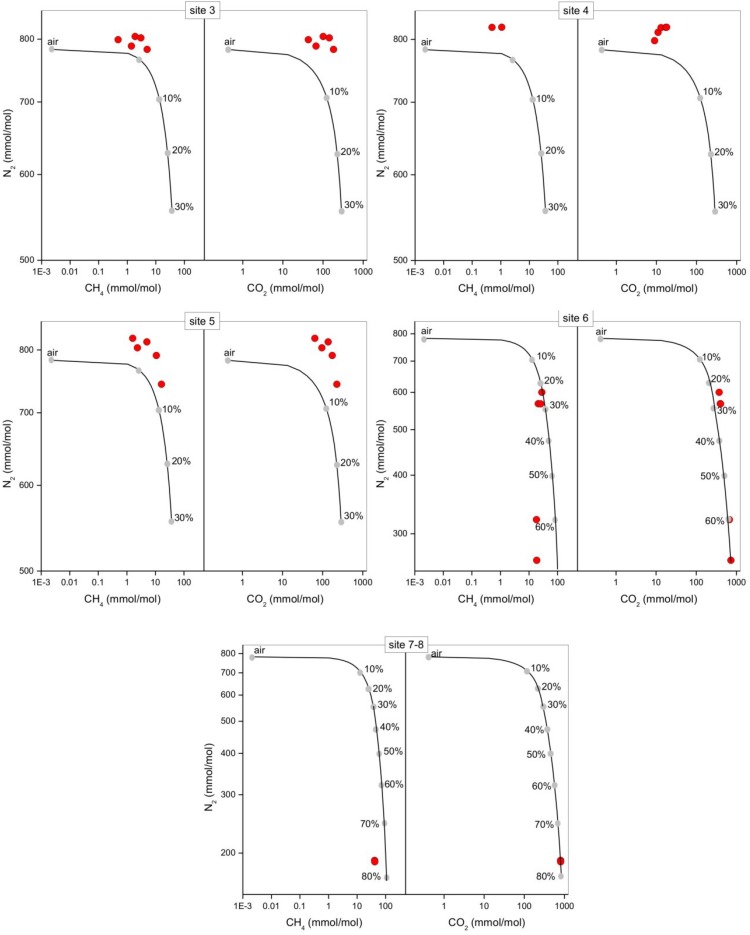
N_2_ vs. CH_4_ and N_2_ vs. CO_2_ binary diagrams for the interstitial soil gasses (red circles) from each sampling site at the different depths (data are shown in Table 2). The expected composition of soil gasses resulting from mixing of air with variable amounts of geogenic gas. The geogenic gas characteristics were obtained by analyzing a free-gas sample collected from one of the bubbling pools (named “BP”). The expected composition of soil gasses then obtained using a simple mass balance modeling approach, is shown (black line and gray dots). The fraction (in percentage) of the deep gas involved in the mixture is also reported.

The bubbling gas was marked by the highest CO_2_ (983 mmol/mol), H_2_S (0.25 mmol/mol), CH_4_ (125μmol/mol) and C_2_H_6_ (7.9 μmol/mol) concentrations and the lowest N_2_ and Ar contents (16 and 1.1 mmol/mol, respectively). The chemical composition of the interstitial soil gasses collected at 10 and 20 cm depth at site 8 resembled that of the gas sample collected at the bubbling pool (BP) (Figure 3). The N_2_ vs. CH_4_ and CO_2_ binary diagrams (Figure 4) show that the interstitial gasses from sites 3 and 4 and those from 10 to 40 cm depth in site 5 were characterized by N_2_ concentrations higher than those expected for air-BP mixing. The interstitial gas sample from site 5 and those collected at 10–30 cm depth from site 6 showed CO_2_ content higher with respect to the air-BP mixing curves. The interstitial soil gasses from sites 6 at depth >30 cm and 8 were depleted in CH_4_ with respect to the air-BP mixing line (Figure 4).

The δ^13^C-CO_2_ values ranged from −11.5 to −6.06‰ vs. V-PDB, showing increasing trends from profiles at site 3 to site 8 and at increasing depth along each profile. The δ^13^C-CO_2_ value of the bubbling gas (−6.41‰ vs. V-PDB) was similar to those of profile at site 8. The δ^13^C-CH_4_ value measured in profile 6 at 20 cm depth was −23.3‰ vs. V-PDB, whereas those at site 8 (at 20 cm depth) and the bubbling gas were −31.7 and −38.5‰ vs. V-PDB, respectively.

### Bacterial and Archaea Abundance and Diversity

**FIGURE 5:**
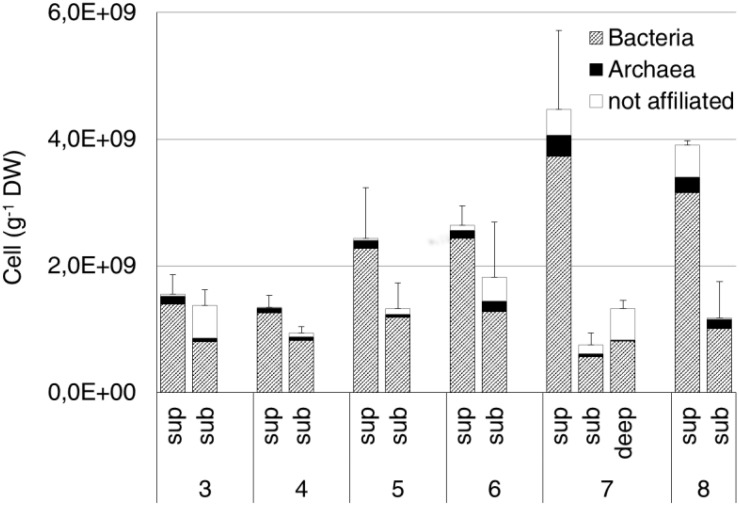
Bacteria and Archaea abundance estimated by CARD-FISH in the original soil samples. Data are expressed as number of cells per gram of dry weight (DW). Cells were hybridized by the specific probes EUB338 I-III and ARCH915 for Bacteria and Archaea, respectively. Error bars represent the standard deviation.

The average prokaryotic abundance showed higher values in the surface (2.7 × 10^9^ ± 1.3 × 10^9^ cell/g DW) than in the subsurface (1.2 × 10^9^ ± 3.7 × 10^8^ cell/g DW) samples, with an overall increase passing from site 3 to sites 7 and 8. The highest abundances were observed at the surface from sites 7 and 8 (4.5 × 10^9^ ± 1.2 × 10^9^ cell/g DW and 3.9 × 10^9^ ± 7.6 × 10^7^ cell/g DW, respectively). The deep sample at site 7 showed an average abundance of 1.3 × 10^9^ ± 1.3 × 10^8^ cell/g DW (Figure 5). Overall, Bacteria (probe EUB338 I-III) represented about 80% of total DAPI stained cells. Archaea (probe ARCH 915) were on average 6% of total cells, with the highest values (12%) measured at site 8 subsurface samples.

The number of OTUs retrieved in each sample was in the range of 1000–1700 and 2200–2600 at sites with high (HC) and low (LC) CO_2_ concentration, respectively. Rarefaction curves show that the LC samples harbor significantly higher diversity than HC samples, regardless of rarefaction depth and the two communities were significantly different from each other (PERMANOVA *p* = 0.002, *R*^2^ = 0.38) (Supplementary Figure 1).

**FIGURE 6:**
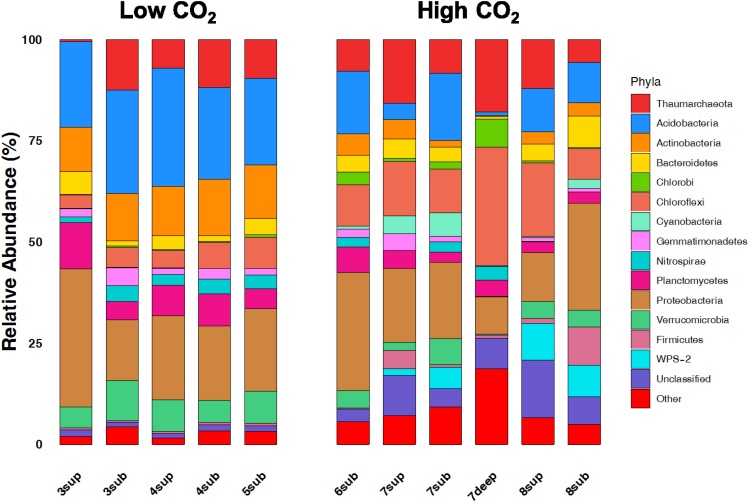
Broad taxonomic affiliation of 16S rRNA gene sequences obtained from environmental samples from high CO_2_ (HC) and low CO_2_ (LC) concentration sites. The relative abundances of the 13 dominant phyla of OTUs belonging to the domain of Bacteria and Archaea are shown as% of total OTUs.

**FIGURE 7:**
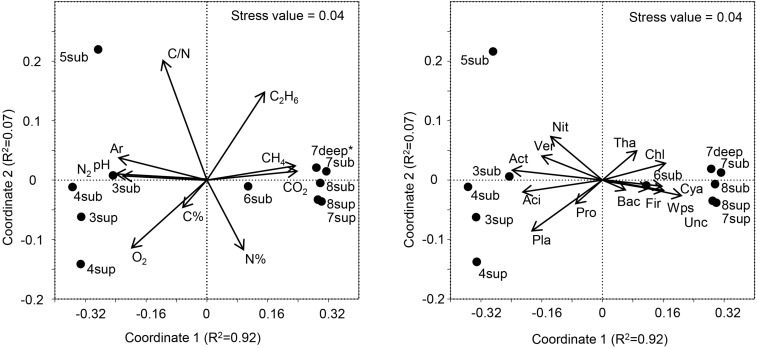
**Left panel:** NMDS ordination plot, based on the Bray Curtis distance matrix, showing the variation patterns of chemical variables. The vector length is proportional to the correlation between the NMDS axes and each chemical variable. **Right panel:** The typifying microbial composition revealed by 16S rRNA gene sequencing projected onto the NMDS ordination synthesizing the chemical dissimilarity between areas. The vector length is proportional to the correlation between the NMDS axes and relative abundance of each microbial phylum. The stress value (value 0.04) provides an accurate representation of the dissimilarity among areas affected or not affected by hydrothermal fluids. Wps, WPS; Chl, Chloroflexi; Ver, Verrucomicrobia; Cya, Cyanobacteria; Aci, Acidobacteria; Act, Actinobacteria; Pla, Planctomycetes; Tha, Thaumarchaeota; Pro, Proteobacteria; Bac, Bacteroidetes; Fir, Firmicutes; Nit, Nitrospira; Unc, Unclassified. ^∗^Gas profile for site 7deep was estimated from the values of sites 7-8sub.

The relative abundances of the 13 dominant phyla are shown in Figure 6, revealing the main differences between microbial communities inhabiting soils at the two CO_2_ concentration levels. Overall, the NMDS ordination plots, showing the variation patterns of the chemical and microbiological variables are reported in Figure 7. Proteobacteria dominated the community in all sampling sites with members within the Xanthomonadaceae (Gammaproteobacteria), being the most abundant family in HC soils. The closest un-cultured match for the most abundant Xanthomonadaceae phylotype is from a sludge reactor for toluene degradation (LC336110, 100% identity).

The second most abundant Proteobacteria in HC samples was within the genus *Geobacter*, whose closest environmental match was from paddy soils (MG101255, 100% identity). This anaerobic genus was already reported in similar environments exposed to high CO_2_ fluxes (Oppermann et al., 2010). Acidobacteria, Actinobacteria, Planctomycetes and Verrucomicrobia showed significantly higher abundances in LC samples. It is worth noting that Chloroflexi, Firmicutes, WPS2 and Cyanobacteria were more abundant in HC samples. Moreover, HC samples showed a higher percentage of undefined sequences when compared to LC samples. The closest environmental match in the NCBI database of the most abundant Unclassified OTU in HC samples is an OTU retrieved in an acid mine drainage (HQ322903, 96% identity).

Chloroflexi classes, Ktedonobacteria and Anaerolineae were abundant only in HC samples. The most abundant Ktedonobacteria OTU, within the Thermogemmatisporaceae family, had a 97% match to an uncultured bacterium from Antarctic soils (EF221335, 97% identity), while the most abundant Anaerolineae phylotype retrieved from HC samples had closest matches with uncultured bacteria from marine sediments (MG637761, 97% identity) and from subfloor sediments in methane hydrate fields (AB540881, 97% identity). Chloroflexi class Dehalococcoidia was only retrieved in one of the HC samples (7deep) and had closest matches with uncultured bacteria from a groundwater aquifer (KC606861, 97% identity) and from sediment from high arsenic groundwater (KF632458, 97% identity).

Thaumarcheota were abundant in all sites except site 3. They made up ∼6% of total community in both LC and HC samples. Two different orders of Thaumarcheota were found in LC and HC samples, respectively: (i) Nitrosospherales (this group also showed to be more abundant in “reference soils (low CO_2_)” (Beulig et al., 2015) (*N. gargensis* and Candidatus Nitrososphaera) and (ii) Cenarcheales (SAGMA-X). The most abundant Nitrosospherales phylotype retrieved from LC samples had a closest match with uncultured bacteria from paddy soils (KP328055, 100% identity), while the closest environmental matches for the most abundant Thaumarchaeota phylotypes in HC samples were from bottled mineral water (JX458345, 99% identity), subtropical forest soil (MH016249, 100% identity) and landfill leachate (KM870444, 100% identity). Our results showed the nearly complete absence from soils of Methanogens that were found only in the most extreme conditions (1.5% only in one HC sample 7deep, data not shown).

Hierarchical clustering for the dataset was generated on the basis of a distance matrix calculated by using the Bray-Curtis distance (Supplementary Figure 2A). This clustering shows a clear profile of the core microbiome with soil samples at high (HC: sites 3-4-5) and low (LC: sites 6sub-7-8) CO_2_ concentrations in different groups. HC samples showed a more dissimilar composition (based on counts on each sample) among each other than the LC samples, as also shown by PCoA plot (Supplementary Figure 2B). The top 10 OTUs that explained the most variance between low and high CO_2_ concentrations according to SIMPER analysis are reported in Table 3 and the mean dissimilarity of the bacterial communities between them was 95%.

**TABLE 3:** Similarity percentage analysis (SIMPER) between environmental samples collected from high CO_2_ (HC) and low CO_2_ (LC) concentration sites.

**OTU ID**	**Phylum**	**Closest known taxonomic classification**	**%Con**	**%Cum**
OTU_1	Thaumarchaeota	Nitrosospheraceae (Family)	2.35	2.35
OTU_3	Thaumarchaeota	Cenarchaeales (SAGMA-X) (Order)	1.69	4.04
OTU_19	Deltaproteobacteria	Syntrophobacteraceae (Family)	1.27	5.31
OTU_12	Chloroflexi	Thermogemmatisporaceae (Family)	1.01	6.33
OTU_6	Gammaproteobacteria	Xanthomonadaceae (Family)	0.94	7.27
OTU_13	Verrucomicrobia	Chthoniobacteraceae (Family)	0.92	8.19
OTU_5	WPS-2		0.72	8.91
OTU_16	Verrucomicrobia	Chthoniobacteraceae (Family)	0.68	9.59
OTU_7	Alphaproteobacteria	Rhizobiales (Order)	0.66	10.24
OTU_68	Acidobacteria	Acidobacteria-6 (Class)	0.63	10.88

*%Con, Contribution; %Cum, Cumulative.*

### Acetogenic Potential

**FIGURE 8:**
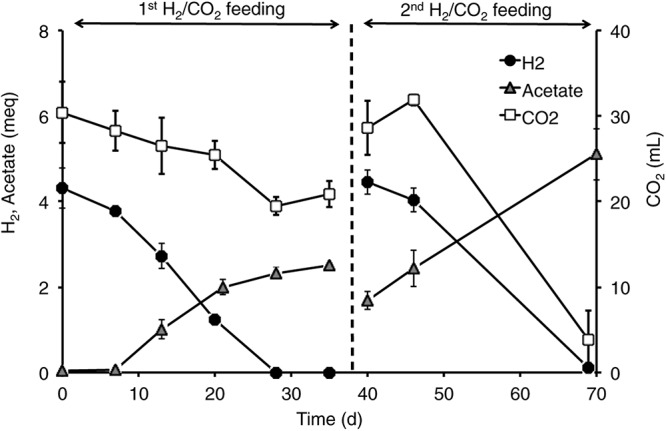
Time course of H_2_, acetate, and CO_2_ in the incubations setup with mofette soil (10 g wet weight in 40 mL anaerobic mineral medium buffered at pH 5.5-6-0) 7sup sampled from a high CO_2_ area. At the start of each feeding cycle, the headspace composition of the bottles consisted of H_2_ (60%, vol/vol), CO_2_ (15%, vol/vol), and N_2_ (25%, vol/vol). The total pressure was around 1 atm. Bottles were incubated statically, in the dark, at room temperature (20–25°C). Error bars represent the standard error of duplicate incubations.

**FIGURE 9:**
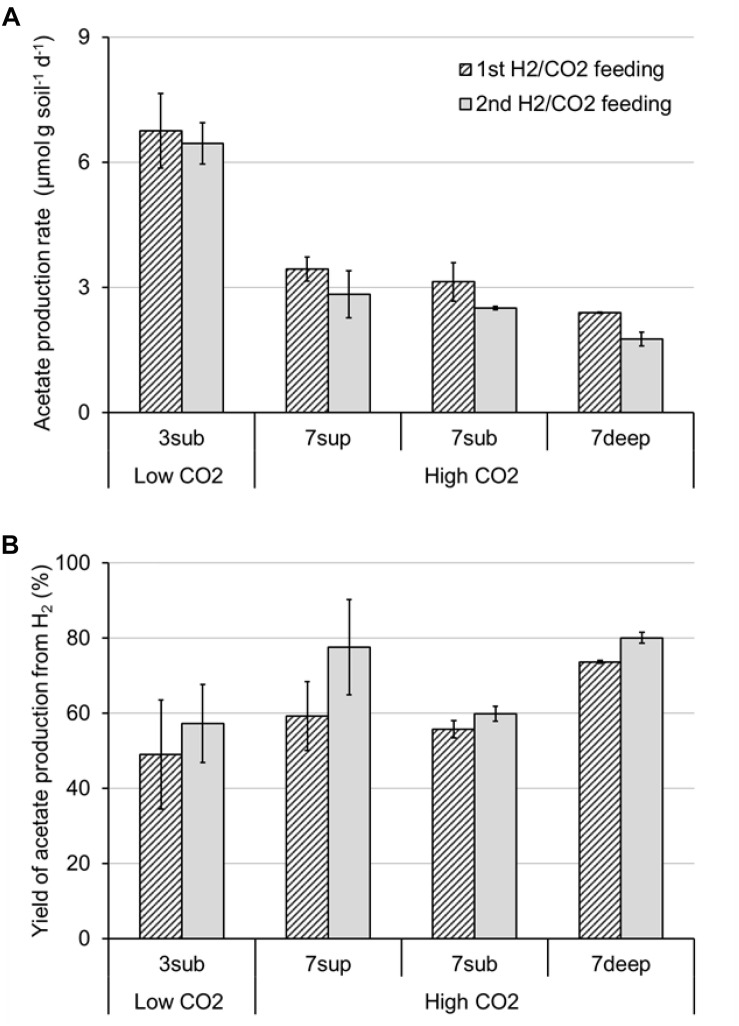
Maximum rate of acetate production **(A)** and yield of acetate production **(B)** from H_2_ in bottles incubated with the different mofette soils (3sub, 7sup, 7sub, and 7deep). Error bars represent the standard error of duplicate incubations.

All the mofette soil incubations supplied with hydrogen gas displayed a remarkable CO_2_ fixation potential, primarily due to the activity of acetogenic (i.e., acetate-producing) microorganisms originally present in the soil. By contrast, negligible production of acetate occurred in control tests incubated with the same soils, under identical conditions, without addition of hydrogen gas. As an example, Figure 8 shows the time course of hydrogen, carbon dioxide, and acetate in a representative incubation experiment setup with soil taken from site 7. Hydrogen and CO_2_ utilization, as well as acetate formation, commenced without any significant lag phase. Upon depletion of hydrogen (on day 28), both acetate production and CO_2_ consumption almost ceased. These latter processes, however, resumed as soon as H_2_ was re-spiked to the bottles (on day 40). Throughout the entire incubation period, the pH remained in the range of 5.5–6.5 and negligible formation of methane and/or of other reduced organic metabolites was detected. Figure 9A compares the observed H_2_-dependent specific acetate formation rates for the different mofette soils. Unexpectedly, the highest values, up to 6.7 ± 0.9 μmol of acetate produced per g of soil (dry weight) per day, were observed in the soil exposed to relatively lower CO_2_ levels. This finding may be due to the lower natural pH of soils exposed to higher CO_2_ levels, which may have resulted in a lower abundance of acetogenic microorganisms. By contrast, the mofette soils exposed to remarkably higher CO_2_ levels displayed a lower and similar acetogenic formation rate, ranging from 1.8 to 3.4 μmol/g ⋅ d. A mass balance indicated that acetate production accounted for 50–80% of consumed H_2_ (Figure 9B), possibly suggesting that other (still not identified) anaerobic H_2_ consuming processes such as sulfate reduction occurred during incubations.

## Discussion

### Origin of Interstitial Gases

The interstitial soil gasses collected at 10 and 20 cm depth at site 8 showed chemical composition similar to that recorded for the gas sample collected from the bubbling pool (BP). The latter, basically dominated by CO_2_ produced at depth by thermometamorphic reactions on carbonates and partially originating from mantle degassing, is to be considered typical of the thermal fluid emission of this area (e.g., Minissale et al., 2002). A moderate increase of the atmospheric inert gasses (N_2_ and Ar), characterizing the interstitial soil gasses with respect to the gas sample from the pool, can likely be related to soil permeability that allowed a significant air contamination of the deep-originated gasses. The atmospheric gas concentrations showed a significant decrease in interstitial gasses from the sites located at decreasing distance from the crater bottom, since air in the soil was counteracted by the flux of the deep-originated gasses, which achieved their highest concentrations in correspondence of the bubbling pool area (Figure 1). Hence, the chemical composition of the interstitial gasses was produced, at a first approximation, by air dilution of the BP-type gas (Figure 3). The consumption of O_2_, a process that typically occurs as fluids circulate underground where reducing conditions are dominating, may have produced an indirect N_2_ increase with respect to that in the air for those samples having a low deep gas contribution such as 3, 4 and 5 (10–40 cm) (Figure 4). The interstitial gas sample from site 5 and those collected at 10–30 cm depth from site 6 show CO_2_ concentrations higher with respect to the curves (Fig. 4) that were constructed considering a simple binary mixing between air and a gas phase having the BP composition. As suggested to explain the indirect N_2_ increase, such a CO_2_-excess was the result of O_2_ depletion affecting the air end-member during its diffusion within the soil, producing a relative enrichment in the other gasses. However, a minor contribution from CH_4_ oxidation cannot be ruled out, as apparently supported by the δ^13^C-CO_2_ values of the interstitial gasses, which were more negative than those of the CO_2_ from the pool, especially those collected at the shallower sampling depths (Table 3). The occurrence of CH_4_ oxidation was confirmed by (i) the composition of the interstitial soil gasses from sites 6 at depth >30 cm and 8 which were depleted in CH_4_ with respect to the air-BP mixing line, and (ii) the δ^13^C-CH_4_ values that were less negative than that of BP, as a result of isotopic fractionation during CH_4_ consumption that typically produces a significant ^13^C-enrichment in the residual CH_4_ (e.g., Tassi et al., 2015; Venturi et al., 2019).

### Microbiome Profiling

The long-term abiotic selection pressure represented by high CO_2_ concentrations is known to drive compositional changes in soil microbial communities (Oppermann et al., 2010; Krüger et al., 2011; Frerichs et al., 2013; Šibanc et al., 2014). However, whether soil CO_2_ concentration itself directly impact soil microbes or whether microbial organisms respond indirectly to co-varying factors such as local hypoxia or elevated soil pH is less clear. O_2_ concentration and pH were among the main co-varying factors that differed between soils with different CO_2_ exposure, both being negatively correlated with CO_2_ (Figure 7). Since O_2_ and pH are major abiotic factors known to affect microbial communities, it is possible that these and not the direct exposure to CO_2_ levels would determine the microbial community composition at the mofette sites (Maček et al., 2011). Nevertheless, it must be considered that the observed variations in both the O_2_ contents and pH were ultimately controlled by the supply of geogenic CO_2_, the latter hindering diffusion of atmospheric O_2_ into the soil and enhancing soil acidification due to increased concentrations of H^+^ and H_2_CO_3_^∗^.

The variation patterns of major physicochemical parameters differentiated those samples positioned intermediately between the less affected by CO_2_ emission (sites 3–5) and the most affected ones (sites 6–8), as revealed by NMDS analysis (Figure 7, Left Panel). The high species richness in the CO_2_-rich soils (1000 to 2600 16S OTUs) puts them on a par with many soils from different environments (Solon et al., 2018). However, the presence of high CO_2_ concentration levels resulted in much lower species richness (16S rRNA gene) and significantly different communities than in sites characterized by lower CO_2_ content, regardless of the depth at which samples were taken. These results corroborate the findings of Beulig et al. (2015), where mofettes exhibited substantially lower prokaryotic diversity than the reference sites. In particular, Chloroflexi, Firmicutes, WPS2 and Cyanobacteria were associated to high CO_2_ conditions, whereas Acidobacteria, Actinobacteria, Planctomycetes and Verrucomicrobia showed higher percentages in Low CO_2_ samples (Figure 7, Right Panel). Most of OTUs retrieved were not identified at the genus level, suggesting that this environment harbors novel bacterial and archaeal diversity, which deserves further investigation to allow fine-scale phylogeny of these communities.

WPS2, initially described in a study on polluted soil in Germany, branch off from either Cyanobacteria or Deinococcus phylum (Nogales et al., 2001). Representatives of this group were already recorded in acidic environments (Grasby et al., 2013; Trexler et al., 2014; Bragina et al., 2015) and a recent study showed that WPS2 is likely an anoxygenic phototroph capable of carbon fixation (Holland-Moritz et al., 2018). Soils exposed to high CO_2_ concentrations could confer an advantage to this group, which would explain its higher abundance relative to lower CO_2_ concentrations.

Chloroflexi have been identified in many environments, including freshwater and marine sediments. Nonetheless, Chloroflexi remain a relatively understudied bacterial lineage. The phylum shows different metabolic lifestyles, including photoautotrophs (e.g., *Chloroflexus aurantiacus*), fermentative (e.g., *Anaerolinea thermophila* UNI-1), organohalide respiring organisms in the Dehalococcoidia, and aerobic thermophiles (e.g., *Thermomicrobium*) (Hug et al., 2013). Although members of Chloroflexi were not directly reported to grow acetogenically, previous studies (e.g., Chan et al., 2013; Hug et al., 2013; Wasmund et al., 2014) suggested that members of Chloroflexi can have the potential to utilize CO_2_ via the acetyl-CoA pathway. Beulig et al. (2015), by SIP analysis of mofette soil incubations, suggested that Chloroflexi perform acetogenesis. Moreover, two Chloroflexi genomes (RBG-2 and RBG-1351), closely related to the Dehalococcoidia, are described to be putative acetogens, utilizing a pathway for the formation of acetate less common in bacteria. For the first time, the complete acetyl-CoA pathway for carbon fixation was described in the Chloroflexi (Hug et al., 2013). In Dehalococcoidia, genes encoding enzymes of the reductive acetyl-CoA pathway were identified, which may enable the fixation of CO_2_ or complete oxidation of organics completely to CO_2_ (Wasmund et al., 2014).

Chloroflexi have been found in high abundance in similar sites exposed to high CO_2_ concentration (Fernández-Montiel et al., 2016; Crognale et al., 2018) and sequences retrieved in this study were affiliated with two classes associated with hypoxic and microhypoxic niches: Ktedonobacteria and Anaerolinea. Ktedonobacteria can thrive in microaerophilic conditions (Chang et al., 2011). Anaerolineae have been reported to grow under strictly anaerobic conditions with isolates found in anaerobic sludge and hot spring (Yamada et al., 2006). Previous studies reported that Ktedonobacteria are prominent in extreme environments such as volcanic, Antarctic, and cave ecosystems (Tebo et al., 2015; Yabe et al., 2017; Schmidt et al., 2018). They comprises only five described species and a large number of uncultured environmental clone sequences (Cavaletti et al., 2006; Yabe et al., 2011; King and King, 2014). Several other strains were isolated, but not formally described, from soil (genus Ktedonobacter), geothermal soils or compost (Stott et al., 2008; Yabe et al., 2011). Moreover, The genome of *K. racemifer* SOSP1-21 T was sequenced and contains a cox operon that confers the potential for carbon monoxide oxidation (Chang et al., 2011; King and King, 2014).

*Anaerolineae* class comprises only a few cultured strains and a large number of environmental 16S rRNA gene sequences. Since Anaerolineae were frequently found within various ecosystems, they were considered to be ubiquitous (Yamada and Sekiguchi, 2009). Two thermophilic, filamentous organisms (i.e., *Anaerolinea thermophila* and *Caldilinea aerophila*) were isolated from an anaerobic granular sludge and a hot spring (Sekiguchi et al., 2003). Moreover, thermophilic and mesophilic filamentous strains were isolated from anaerobic sludge blanket at a temperature range of 25–50°C (Yamada et al., 2005) and in hydrothermal vents in the Yaeyama Archipelago in Japan (Nunoura et al., 2013).

Acidobacteria were more abundant in low CO_2_ samples than in those exposed to high CO_2_ fluxes, a pattern previously reported in similar environments (Crognale et al., 2018). Members of the phylum Acidobacteria represent one of the predominant bacterial groups in soil but their ecological functions are still poorly understood (Jones et al., 2009; Foesel et al., 2014; Kielak et al., 2016). Acidobacteria were abundantly found in volcanic craters (Glamoclija and Garrel, 2004; Crognale et al., 2018), in marine vents (Sievert et al., 2000; López-García et al., 2003) and in the Yellowstone National Park geothermal areas (Norris et al., 2002).

In both LC and HC samples (except site 3), the archaeal OTUs were mainly affiliated to Thaumarchaeota, a deep-branching phylum within Archaea domain. An increase in Thaumarchaeota associated sequences was recorded in other mofettes with high CO_2_ concentrations (Frerichs et al., 2013; Šibanc et al., 2014; Crognale et al., 2018) and it was proposed that this could be an “indicator taxa” of high CO_2_ enriched soils (Frerichs et al., 2013), which could be connected to the CO_2_ soil acidification and the obligate acidophilic nature of these taxa (Lehtovirta-Morley et al., 2011). Thaumarchaeota are among the most abundant Archaea on Earth and are believed to significantly contribute to the global N-cycle and C-cycle. This phylum includes not only all known Ammonia-Oxidizing Archaea (AOA) but also several clusters with unknown energy metabolism (Pester et al., 2011; Stieglmeier et al., 2014). Previous studies suggested that ammonia-oxidizing Thaumarchaeota can be adapted to both low ammonia availability and an autotrophic or mixotrophic lifestyle (Zhang et al., 2008; Schleper and Nicol, 2010; Pratscher et al., 2011). It has also been suggested that members of this phylum may be involved in methane oxidation (Crognale et al., 2018) since ammonia monooxygenase and methane monooxygenase enzymes are evolutionarily linked (Holmes et al., 1995), enabling both methanotrophy and ammonia oxidation (O’Neill and Wilkinson, 1977; Jones and Morita, 1983). The presence of the two different clades in different CO_2_ concentrations, Nitrosospherales and Cenarcheales in LC and HC, respectively, suggests strong selection by local environmental conditions: Cenarcheales found at high CO_2_ may not be ammonia-oxidizers and have a different metabolic pathway for energy production or may have extremely high affinity for O_2_, which allows their survival as ammonia oxidizers at very high CO_2_ concentrations. At higher O_2_ concentrations they may be outcompeted by Nitrosospherales. The circadian Oxygen oscillation could also justify the presence of ammonia-oxidizing archaea. Interestingly, Methanobacteria class, belonging to the South African Gold Mine Euryarchaeotic Group (SAGMEG), was only found in the most extreme conditions (1.5% only in the sample 7deep). OTUs affiliated with archaeal groups that include methanogens were nearly absent from this site, as previously reported in similar acidic soils exposed to natural CO_2_ fluxes (Crognale et al., 2018). The nearly complete absence of methanogens in the study soils could be due to the fact that these microorganisms were outcompeted by either acetogens, as also discussed in the following paragraph, or the fluctuating oxygen concentration during daytime. This suggests that methanogenesis marginally contributes to the overall metabolic features of the microbial communities in such an environment.

### Acetogenic Bacteria

Acetogenic bacteria are ubiquitous in nature and are a specialized group of strictly anaerobic bacteria.

Acetogens were isolated from diverse environments, including soils, hypersaline waters and sediments (Liu and Conrad, 2011). In these ecosystems, chemolithoautotrophic acetogenic bacteria can directly compete with methanogenic archaea (hydrogenotrophic) or syntrophically interact with acetoclastic methanogens (Chassard and Bernalier-Donadille, 2006; Liu and Conrad, 2011). Acetogens include Firmicutes, Spirochaetes, Delta-Proteobacteria and Acidobacteria (Drake et al., 2006). Recently Beulig et al. (2015) speculated that Chloroflexi might be involved in acetogenesis in wetland mofette. Comparing the community composition in the environmental samples from sites at low (LC) and at high CO_2_ concentration (HC), our results suggest a major role of Acidobacteria in LC sites whereas in HC sites Chloroflexi and Firmicutes are likely the predominant acetogens. This could also explain the difference in yield of acetate production observed in the incubation experiments with soil from low and at high CO_2_ concentration sites. Under standard conditions, H_2_-CO_2_-dependent methanogenesis (ΔG^o^’ = −130 kJ/reaction) is more energetically favorable than H_2_-CO_2_-dependent acetogenesis (ΔG^o^’ = −95 kJ/reaction). At low H_2_ concentrations, typically found in many anoxic environments, methanogenesis is energetically more favorable than acetogenesis, and hydrogenotrophic methanogens outcompete chemolithoautotrophic acetogens (Drake et al., 2006). However, acetogens have a kinetic advantage over methanogens at acidic pH and low temperature (Conrad et al., 1989; Kotsyurbenko et al., 1993), hence providing a likely explanation for the remarkable acetogenic potential observed in the incubation experiments, together with the lack of methanogenic activity, further confirmed by the absence of methanogens in sequencing analyses. In spite of that, it should be considered that the herein described incubation tests would have likely resulted in the selective enrichments of acetogens (e.g., Firmicutes), thereby overestimating the actual acetogenic potential of the natural communities.

## Concluding Remarks

Gas discharges, characterized by extremely high CO_2_ concentrations, are significantly affecting soil formation processes interacting with the geomorphological gradient observed in the mofette. Consequently, low pH values at the bottom of the Bossoleto sinkhole, as well as increased organic carbon accumulation in the topsoil and removal of inorganic carbon, were recorded. Similarly, variations in macro and micro-elements concentrations were also observed, likely due to the effect of the pH on element mobility. The comparison between interstitial soil gasses and those collected in an adjacent bubbling pool and the isotopic carbon fractionation, clearly indicated an increase in CO_2_ due to CH_4_ oxidation at the bottom of the sinkhole. The extremely high CO_2_ concentrations resulted in higher microbial abundance and a lowered microbial diversity by favoring bacteria already reported to be involved in acetogenesis in mofette soils (i.e., Chloroflexi, Acidobacteria and Firmicutes). All the mofette soils supplied with hydrogen gas, in experimental incubations, displayed a remarkable CO_2_ fixation potential, primarily due to the activity of acetogenic (i.e., acetate-producing) microorganisms. By contrast, negligible production of acetate occurred in control tests incubated with the same soils, under identical conditions, without the addition of H_2_. Our results suggest that the composition of microbial communities at high CO_2_ concentrations affects carbon cycling through inhibition of organic carbon decomposition and increases CO_2_-fixation via the acetyl-CoA pathway. These acetogenic organisms, outcompeting methanogens, might determine considerable changes in carbon cycling leading to the accumulation of organic carbon in soils at the bottom of the mofette. Sites naturally exposed to extremely high CO_2_ levels could, therefore, potentially represent an untapped source of microorganisms with unique capabilities to catalytically convert CO_2_ into valuable organic chemicals for industrial applications.

## Data Availability Statement

The 16S rRNA gene sequences from Illumina MiSeq libraries from this study were deposited in the SRA (Short Read Archive) database under Bioproject ID PRJNA548940.

## Author Contributions

SF conceived the study. SF, FUn, FT, OV, AR, and FA contributed to the conception and design of the study. All authors performed the sampling campaign and the field and laboratory analysis, wrote the sections of the manuscript, contributed to manuscript revision, and read and approved the submitted version. LV and SV organized the database and performed the statistical analysis. SF wrote the first draft of the manuscript.

## Conflict of Interest

The authors declare that the research was conducted in the absence of any commercial or financial relationships that could be construed as a potential conflict of interest.
