# Supplementary material for: Microbiomes in Soils Exposed to Naturally High Concentrations of CO2 (Bossoleto Mofette Tuscany, Italy)
Source: Front Microbiol. 2019 Oct 4;10:2238. doi: 10.3389/fmicb.2019.02238 (PMC6797827; doi:10.3389/fmicb.2019.02238)
Supplement: Supplementary file 1 [file Table_1.docx]

Microbiomes in soils exposed to naturally high concentrations of CO_2_ (Bossoleto mofette Tuscany, Italy)

Fazi S., Ungaro F., Venturi S., Vimercati L, Cruz-Viggi C., Baronti S., Ugolini F., Calzolari C., Tassi F., Vaselli O., Raschi A., Aulenta F.

**Supplementary material**

**Supp. Table 1 -** Chemical composition resulting from ICP-OES analysis of soil. Data are expressed in ppm.

|  | **Site 3** | **Site 4** | **Site 7** |
| --- | --- | --- | --- |
| Al | 20040.0 | 19790.0 | 22810.0 |
| Ba | 81.1 | 95.7 | 178.5 |
| Ca | 77430.0 | 90250.0 | 5505.0 |
| Cd | 2.2 | 2.0 | 3.9 |
| Co | 6.8 | 6.1 | 4.4 |
| Cr | 42.2 | 40.0 | 69.4 |
| Cu | 14.5 | 16.2 | 82.3 |
| Fe | 17280.0 | 14940.0 | 31540.0 |
| K | 1729.0 | 2107.0 | 4087.0 |
| Li | 24.2 | 16.0 | 7.6 |
| Mg | 4418.0 | 4538.0 | 2548.0 |
| Mn | 882.7 | 794.7 | 274.1 |
| Na | 214.3 | 307.8 | 240.3 |
| Ni | 27.3 | 24.0 | 19.3 |
| Ni | 24.4 | 21.7 | 14.8 |
| P | 616.8 | 869.1 | 5501.0 |
| Pb | 23.1 | 24.8 | 124.9 |
| Sr | 525.8 | 742.6 | 95.8 |
| Zn | 56.5 | 53.2 | 80.4 |
