# Supplementary material for: Microbiomes in Soils Exposed to Naturally High Concentrations of CO2 (Bossoleto Mofette Tuscany, Italy)
Source: Front Microbiol. 2019 Oct 4;10:2238. doi: 10.3389/fmicb.2019.02238 (PMC6797827; doi:10.3389/fmicb.2019.02238)
Supplement: Supplementary file 2 [file Image_1.pdf]

Microbiomes in soils exposed to naturally high concentrations of CO_2 (Bossoleto mofette Tuscany, Italy)

Supplementary material

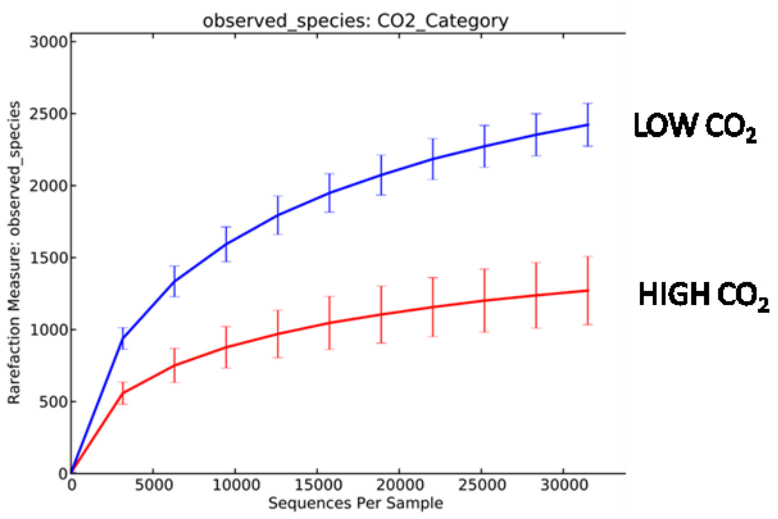

Supp. Fig. 1 - Alpha rarefaction curves based on the number of OTUs observed of 16S rRNA gene sequences obtained from soil samples at high (HC) and low (LC) CO_2 concentrations in the Bossoleto sinkhole. Data shown are means ± s.e. The number of bacterial OTUs observed in HC samples is about half of the number of bacterial OTUs observed in LC samples. The metric reveals that sampling saturation is reached at a smaller sequencing depth for HC samples compared to LC samples.
