# Supplementary material for: Microbiomes in Soils Exposed to Naturally High Concentrations of CO2 (Bossoleto Mofette Tuscany, Italy)
Source: Front Microbiol. 2019 Oct 4;10:2238. doi: 10.3389/fmicb.2019.02238 (PMC6797827; doi:10.3389/fmicb.2019.02238)
Supplement: Supplementary file 3 [file Image_2.pdf]

Microbiomes in soils exposed to naturally high concentrations of CO_2 (Bossoleto mofette Tuscany, Italy)

Supplementary material

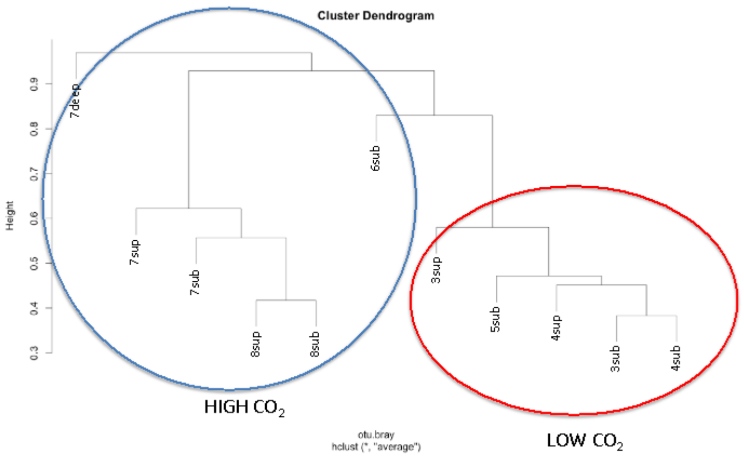

A

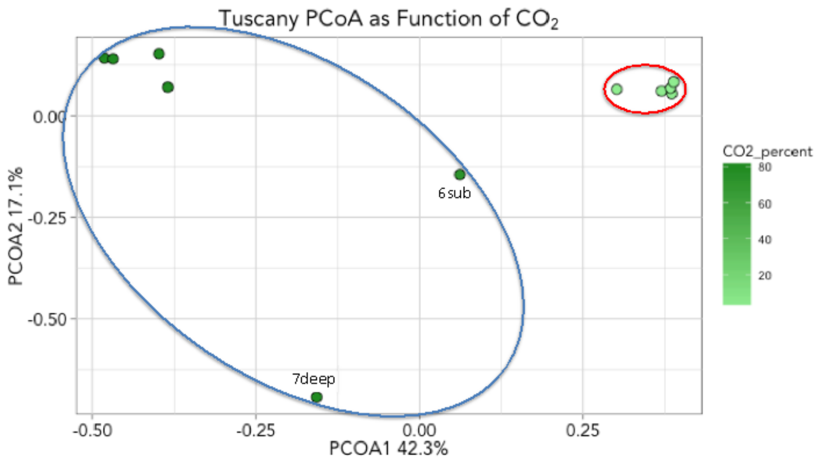

B

Supp. Fig. 2 - A: Hierarchical clustering of HC and LC soil samples from the Bossoleto sinkhole based on a distance matrix calculated by using the Bray-Curtis distance. HC and LC samples cluster in different groups, with HC samples showing a more dissimilar composition (based on counts on each sample) among each other than LC samples.

B: Cluster diagram-based PCoA plot using Bray Curtis distance and Hellinger transformed of bacterial soil communities of the Bossoleto sink hole. Ovals surround total HC and LC communities. HC soil communities and LC soil communities are significantly different from each other (PERMANOVA p = 0.002, R^2 = 0.38). Conversely, HC and LC samples did not cluster by depth (PERMANOVA p = 0.328, R^2 = 0.10).
